# Consolidative thoracic radiotherapy improves the prognosis of extensive stage small-cell lung cancer patients in the chemoimmunotherapy era: a multicenter retrospective analysis

**DOI:** 10.1080/07853890.2025.2542434

**Published:** 2025-08-04

**Authors:** Nan Yao, Shuai Li, Lingling Hu, Yixian Pei, Zhaohui Qin, Na Li, Shaodong Tong, Nan Zhang, Yuanhu Yao

**Affiliations:** a Department of Radiation Oncology, The Affiliated Wuxi People’s Hospital of Nanjing Medical University, Wuxi, Jiangsu, China; b Wuxi Medical Center, Nanjing Medical University, Wuxi, Jiangsu, China; c Wuxi People’s Hospital, Wuxi, Jiangsu, China; d Department of Radiation Oncology, Affiliated Hospital of Jiangnan University, Wuxi, Jiangsu, China; e Department of Hematology, Ruijin Hospital Wuxi Branch, Shanghai Jiao Tong University School of Medicine, Wuxi, Jiangsu, China; f Research Center for Medical and Health Emergency Rescue, Xuzhou Medical University, Xuzhou, Jiangsu, China; g Department of Radiation Oncology, Xuzhou Central Hospital, Xuzhou, Jiangsu, China; h Department of Radiation Oncology, Xuzhou Cancer Hospital, Xuzhou, Jiangsu, China; i Department of Pulmonary and Critical Care Medicine, The Affiliated Wuxi People’s Hospital of Nanjing Medical University, Wuxi People’s Hospital, Wuxi, Jiangsu, China

**Keywords:** Chemoimmunotherapy, consolidative thoracic radiotherapy, extensive-stage small cell lung cancer, efficacy, safety

## Abstract

**Background:**

For extensive-stage small cell lung cancer (ES-SCLC) with intrathoracic residuals after chemotherapy, the landmark CREST trial demonstrated the benefit of consolidative thoracic radiotherapy (cTRT). Yet the efficacy and safety of cTRT after chemoimmunotherapy for ES-SCLC remain largely unknown. This study aimed to assess the role of cTRT following chemoimmunotherapy in patients with ES-SCLC.

**Methods:**

A retrospective analysis of ES-SCLC patients without disease progression after first-line chemoimmunotherapy was conducted between March 2019 and November 2021. Based on whether cTRT or not, patients were allocated to cTRT group or non-cTRT group. We evaluated efficacy by using the median overall survival (mOS) and progression-free survival (mPFS) times, and safety by measuring the incidence of adverse events.

**Results:**

During this study, 72 patients with ES-SCLC were enrolled, with a median follow-up of 34.66 months. Twenty-nine patients received cTRT and 43 patients did not receive cTRT. Among the cTRT group and the non-cTRT group, the mPFS was 11.50 and 8.02 months, respectively, with a HR of 0.60 (95% CI 0.36–0.99, *p* = 0.043). The mOS in the cTRT group was also significantly longer than that in the non-cTRT group (28.68 months vs. 16.30 months, HR = 0.56, 95% CI 0.32–0.96, *p* = 0.033). Based on multivariate analysis, cTRT and cycles of immunotherapy ≥ 6 were independent factors affecting survival. There were no treatment-related deaths and most adverse events were grade 1–2.

**Conclusions:**

This study suggests that the addition of cTRT to first-line chemo­immunotherapy significantly improves survival in ES-SCLC with well-tolerated toxicity.

## Key Messages

- There is a paucity of evidence regarding the efficacy and safety of consolidative thoracic radiotherapy (cTRT) after chemoimmunotherapy for ES-SCLC.
- We conducted a real-world study to investigate whether cTRT is necessary in patients with ES-SCLC who responded well to chemoimmunotherapy.
- ES-SCLC patients treated with cTRT after first-line chemoimmunotherapy had prolonged PFS and OS with an acceptable safety profile in our study.

## Introduction

Approximately 13% to 17% of lung cancers are small-cell lung cancers (SCLC), a particularly malignant neuroendocrine carcinoma that has an aggressive growth pattern and a tendency to spread widely at an early stage in the disease [1,2]. Extensive-stage SCLC (ES-SCLC) accounts for approximately two-thirds of all newly diagnosed SCLC cases and has a poor prognosis [3]. A platinum-based doublet chemotherapy has become the standard of care for the treatment of ES-SCLC over the past few decades. However, 75% of patients have residual thoracic lesions that typically recur within one year [4]. In the era of chemotherapy, the CREST study demonstrated that for ES-SCLC patients who responded well to chemotherapy, consolidative thoracic radiotherapy (cTRT) could raise local control rates and improve overall survival (OS) [5].

Recently, with the emergence of immune checkpoint inhibitors, survival for ES-SCLC has improved to over one year, and chemoimmunotherapy has become the first-line treatment, but only 0.8%–2.5% presented a complete response [6]. Even with consolidation immunotherapy, residual lesions commonly progress rapidly, with a median progression-free survival (mPFS) of 5–6 months. Improved local control and overall survival are urgently required through the development of new treatment modes.

Increasing evidence suggests a possible synergistic effect between radiotherapy and immunotherapy. Besides mediating cancer cell death induced by DNA damage, radiotherapy also modulates tumour immunogenicity by triggering proinflammatory mediator release, stimulating immune cells to infiltrate tumours, and enhancing neoantigen expression [7,8]. Moreover, it may promote systemic antitumor immunity, leading to the control of distal metastases; this phenomenon is known as the abscopal effect [9–11].

However, controversy continues to surround the role of cTRT in ES-SCLC following chemoimmunotherapy. The main reason is the lack of incorporation of cTRT after chemoimmunotherapy in critical ES-SCLC studies, including the IMpower133, CASPIAN, ASTRUM005, and CAPSTONE-1 trials [6,12–14]. Furthermore, there are concerns regarding the increased risk of pneumonitis, as well as uncertainty regarding the magnitude of the benefit from this combination of approaches. In order to assess the efficacy and safety of cTRT following first-line chemoimmunotherapy in patients with ES-SCLC, we performed this real-world study and further explored the factors that may influence its efficacy.

## Methods

### Patients

Between March 2019 and November 2021, ES-SCLC patients who were treated with first-line chemoimmunotherapy followed by immunotherapy maintenance (with or without cTRT) were retrospectively identified at four institutions (The Affiliated Wuxi People’s Hospital of Nanjing Medical University, The Affiliated Hospital of Xuzhou Medical University, Xuzhou Cancer Hospital, and Xuzhou Central Hospital). The patient data, clinical features, pathologic and molecular reports, treatment details, and follow-up outcomes were retrieved from the electronic medical records of the hospitals and collated.

Inclusion criteria for patient selection were: 1) an age of at least 18 years, 2) cytologically or histologically confirmed as SCLC, 3) a diagnosis of ES-SCLC according to the Veterans Administration Lung Study Group staging system, and 4) obtaining a good response (complete or partial response and stable disease, according to Response Evaluation Criteria in Solid Tumors [RECIST] criteria [15]) to first-line chemoimmunotherapy. Exclusion criteria were as follows: 1) complicated with other malignancies, 2) disease progression after initial chemoimmunotherapy, 3) without complete data. Patients were assigned to a cTRT group or a non-cTRT group depending on whether they underwent cTRT. This study complied with the Declaration of Helsinki and was approved by the ethics committee of Wuxi People’s Hospital (KY22117). The need to obtain informed consent was waived due to the retrospective nature of this study.

### Treatment approach

All patients were treated with immunotherapy plus platinum-etoposide chemotherapy every 3 weeks up to 6 cycles. The administration of concurrent immunotherapy usually commences on the first day of a treatment cycle and before the administration of chemoagents. Maintenance immunotherapy was administered every three weeks until the disease progressed or unacceptable toxic effects were observed. Treatment with cTRT was determined at the discretion of the treating physician using intensity modulation radiation therapy. Gross target volume included the residual primary tumor after chemoimmunotherapy and positive lymph nodes. All positive nodal regions at diagnosis were included in the clinical target volume, which was expanded from the gross target volume with a margin of 0.8 cm. There was a 0.50 cm increase in the planning target volume compared to the clinical target volume.

### Endpoints and assessment

PFS, OS, and safety were the primary endpoints. RECIST 1.1 was used to evaluate clinical response to treatment. PFS was calculated from the date of diagnosis to disease progression (or last follow-up or death). OS was calculated from the date of diagnosis until the date of death or the last follow-up (censored). Toxicities were graded in accordance with the Common Terminology Criteria for Adverse Events (AEs) version 5.0 [16]. Patients were required to undergo a clinical visit and a computed tomography scan every 2 to 3 months for evaluation of toxicity and clinical response.

### Statistical analysis

Demographics and clinical characteristics were compared between patients given cTRT and non-cTRT. The Chi-squared and Fisher’s exact tests were applied to categorical variables, and the Student’s t tests were applied to continuous variables. Median follow-up was calculated with the inverse Kaplan–Meier method. To compare the survival differences between the two treatment groups, a Kaplan–Meier survival curve and log-rank test were used. To assess the association between clinicopathologic characteristics and survival outcomes, univariate and multivariate analyses were performed. *P* values <0.05 were taken as statistically significant. SPSS Statistics version 26.0 (SPSS Inc., Chicago, IL, USA) and R software (version 4.2.1) were used to perform all statistical analyses.

## Results

### Patients characteristics and treatment exposure

**Figure 1.**
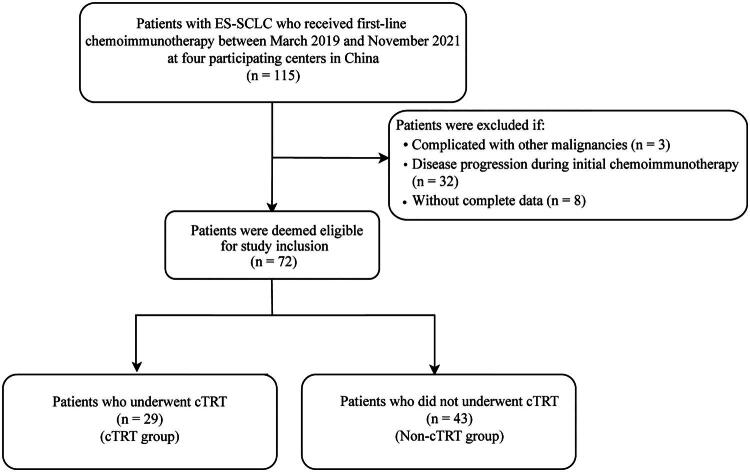
Diagram of study patients’ identification. ES-SCLC, extensive-stage small cell lung cancer; cTRT, consolidative thoracic radiotherapy.

After implementing the inclusion and exclusion criteria, 72 patients were finally enrolled in this study, 29 of whom underwent cTRT and 43 did not (Figure 1). The clinical characteristics were well-balanced in the two groups (Table 1). The median age of our study population was 63 years (range 34–82 years). For the whole cohort, most patients were male (75.00%), Eastern Cooperative Oncology Group performance status (ECOG PS) 0–1 (87.50%), smoker (58.33%), extrathoracic metastasis (63.89%), number of initial distant metastases <3 (72.22%), with partial response to initial treatment (91.67%), and without prophylactic cranial irradiation (PCI) (73.61%).

**Table 1.** Baseline clinical and demographic characteristics of all enrolled patients with ES-SCLC.

Characteristic	cTRT (*n* = 29)	Non-cTRT (*n* = 43)	*p* value
Age, median (range), years	63 (34–80)	65 (49–82)	0.14
<65	17 (58.62)	21 (48.84)	0.42
≥65	12 (41.38)	22 (51.16)	
Sex, *n* (%)			0.33
Male	20 (68.97)	34 (79.07)	
Female	9 (31.03)	9 (20.93)	
ECOG PS, *n* (%)			0.41
0–1	27 (93.10)	36 (83.72)	
2	2 (6.90)	7 (16.28)	
Smoking status, *n* (%)			0.60
Never	11 (37.93)	19 (44.19)	
Previous or current	18 (62.07)	24 (55.81)	
Extrathoracic metastasis, *n* (%)			0.81
Yes	19 (65.52)	27 (62.79)	
No	10 (34.48)	16 (37.21)	
Number of initial distant metastases, *n* (%)			0.57
<3	22 (75.86)	30 (69.77)	
≥3	7 (24.14)	13 (30.23)	
Response to initial treatment, *n* (%)			0.94
Complete	3 (10.34)	3 (6.98)	
Partial	26 (89.66)	40 (93.02)	
Cycles of immunotherapy, *n* (%)			0.60
<6	13 (44.83)	22 (51.16)	
≥6	16 (55.17)	21 (48.84)	
Prophylactic cranial irradiation, *n* (%)			0.07
Yes	11 (37.93)	8 (18.60)	
No	18 (62.07)	35 (81.40)	

ES-SCLC, extensive-stage small cell lung cancer; cTRT, consolidative thoracic radiotherapy; ECOG-PS, Eastern Cooperative Oncology Group Performance Status.

Fifty-six patients (77.78%) received an anti-programmed cell death-ligand 1 agent (durvalumab: 13 in the cTRT group and 17 in the non-cTRT group or atezolizumab: 11 in the cTRT group and 15 in the non-cTRT group), and only 16 (22.22%) received an anti-programmed cell death protein 1 agent (tislelizumab: 3 in the cTRT group and 5 in the non-cTRT group or serplulimab: 2 in the cTRT group and 6 in the non-cTRT group). The median number of immunotherapy cycles (including maintenance therapy) was 6 (range, 4–23) in the cTRT group and 5 (range, 3–28) in the non-cTRT group. In the cTRT group, almost all patients underwent sequential cTRT in the interval of two cycles of immunotherapy, but only two patients underwent concurrent immunotherapy and cTRT. The most common cTRT regimens were 30 Gy in 10 fractions (59.72%) followed by 50 Gy in 25 fractions (22.22%) and 60 Gy in 30 fractions (5.56%). Among 19 patients who underwent PCI, 25 Gy in 10 fractions was the most frequently used scheme. A total of 22 patients (30.56%) received radiotherapy for the palliation of metastatic disease in addition to TRT or PCI.

### Survival analysis

**Figure 2.**
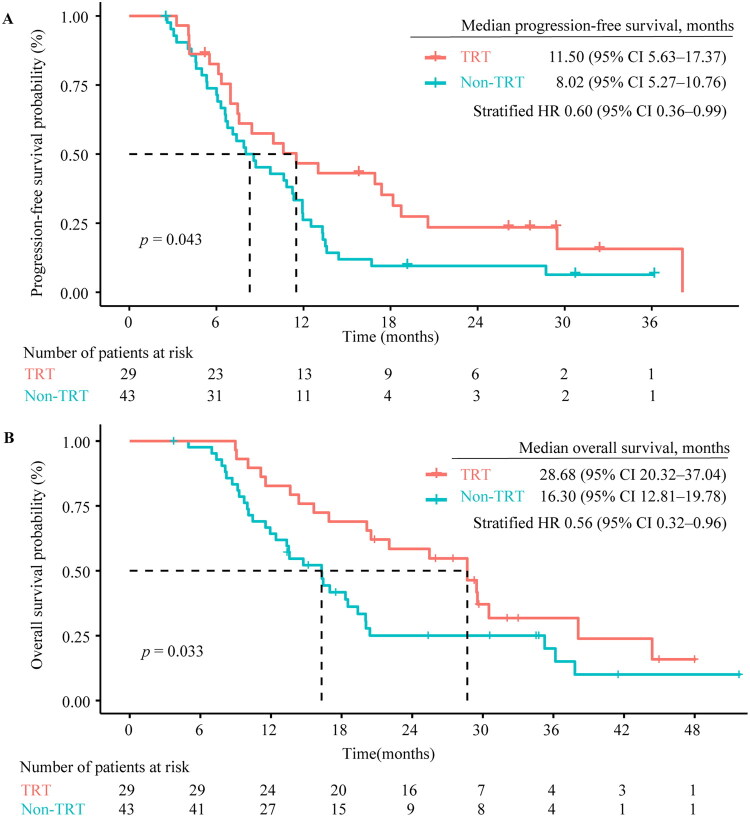
Kaplan–Meier plots of progression-free survival (A) and overall survival (B). HR, hazard ratio.

At the data cut-off date of 11 April 2024, the median follow-up time was 34.66 months. In the cTRT group, the median PFS was 11.50 months in comparison with 8.02 months in non-cTRT groups (hazard ratio [HR] = 0.60, 95% confidence interval [CI] 0.36–0.99, *p* = 0.043; Figure 2A). PFS rates were 79.0% versus 71.4% and 46.7% versus 26.2% in the two groups at 6 months and 12 months, respectively. Patients who received cTRT had a significantly longer OS than those who did not, with mOS of 28.68 and 16.30 months, respectively, HR = 0.56 (95% CI 0.32–0.96, *p* = 0.033; Figure 2B). OS rates were 82.8% versus 64.3% and 69.0% versus 39.0% for the two groups at 12 and 18 months.

Table 2 summarizes analysis of univariate and multivariate Cox proportional hazards regressions of OS prognostic factors. According to univariate analyses, extrathoracic metastasis, immunotherapy cycles ≥6, and cTRT were significant prognostic factors for OS. Using these three characteristics as parameters, multivariate analysis revealed that cycles of immunotherapy ≥6 and cTRT were positively associated with OS. However, extrathoracic metastasis did not have a significant effect.

**Table 2.** Univariate and multivariate analyses of factors influencing overall survival.

Variables	Univariate analyses		Multivariate analyses	
	HR (95% CI)	*p*	HR (95% CI)	*p*
Age (<65 vs. ≥65; years)	1.20 (0.70–2.08)	0.504		
Sex (Male vs. Female)	1.72 (0.90–3.29)	0.10		
ECOG PS (0–1 vs. 2)	0.58 (0.26–1.30)	0.19		
Smoking status (Never vs. Previous or current)	0.78 (0.45–1.35)	0.37		
Extrathoracic metastasis (Yes vs. No)	1.92 (1.05–3.57)	0.04	0.56 (0.30–1.03)	0.06
Number of initial distant metastases (<3 vs. ≥3)	0.75 (0.42–1.36)	0.35		
Cycles of immunotherapy (<6 vs. ≥6)	1.88 (1.09–3.24)	0.02	1.87 (1.08–3.25)	0.03
cTRT (Yes vs. No)	0.56 (0.32–0.96)	0.03	0.56 (0.32–0.99)	0.046
PCI (Yes vs. No)	0.62 (0.32–1.20)	0.16		

HR, hazard ratio; CI, confidence interval; ECOG PS, Eastern Cooperative Oncology Group performance status; cTRT, consolidative thoracic radiotherapy; PCI, Prophylactic cranial irradiation.

**Figure 3.**
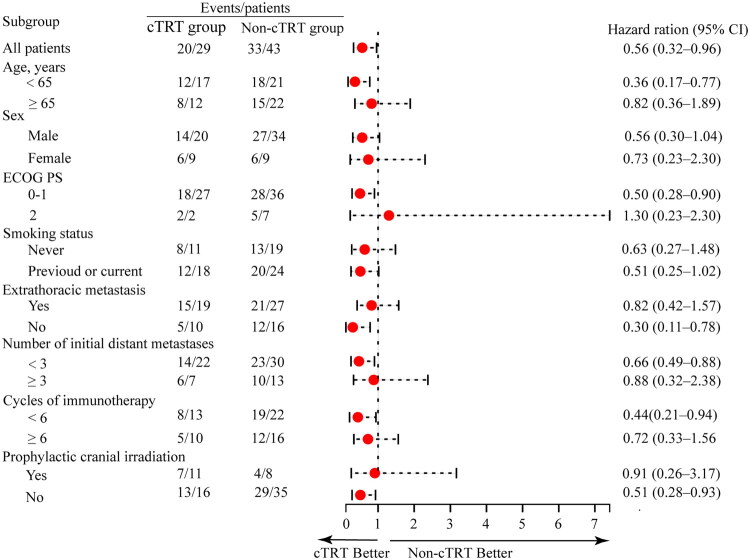
Subgroup analysis for overall survival. cTRT, consolidative thoracic radiotherapy; CI, confidence interval; ECOG PS, Eastern Cooperative Oncology Group performance status.

Exploratory subgroup analyses in our study showed that significantly longer OS were observed among groups including age <65 years, ECOG PS of 0–1, without extrathoracic metastasis, number of initial distant metastases <3, Cycles of immunotherapy <6, and without PCI (Figure 3).

### Disease progression patterns

The local response to cTRT was shown in Supplementary Table S1. At the time of data cut-off, 62 patients (86.11%) experienced disease progression, including 23 in the cTRT group and 39 in the non-cTRT group. Intrathoracic progression occurred at a rate of 21.74% (5/23) in the cTRT group and 48.72% (19/39) in the non-cTRT group (*p* = 0.04). Brain and bone were the most common sites of distant metastasis.

### Toxicity

The incidence of treatment-related AEs in all patients was summarized in Table 3. There were no treatment-related deaths observed in our cohort up to the cut-off date. AEs incidence did not differ significantly between the two groups. The major Grade 3 or higher AEs was neutrophil count decreased, white blood cell count decreased, and anemia in the two groups. In cTRT group, Grade 3 or 4 pneumonitis and esophagitis both occurred in only one case and improved after symptomatic treatment. The patient with Grade 3 esophagitis improved after supportive care included analgesia, anti-infection, and intravenous nutrition. After symptomatic supportive treatment including oxygen inhalation, phlegm and asthma relief, glucocorticoid therapy with gradually reduced dosage and discontinuation of immunotherapy, patients with Grade 3 or 4 pneumonitis in the two groups recovered. Other low-grade treatment-related adverse events were listed in Supplemental Table S2.

**Table 3.** Incidence of treatment-related adverse events in ES-SCLC patients.

Event, *n* (%)	All grades		Grade 3 or higher	
	cTRT (*n* = 29)	Non-cTRT (*n* = 43)	cTRT (*n* = 29)	Non-cTRT (*n* = 43)
Pneumonitis	4 (13.79)	3 (6.98)	1 (3.45)	1 (2.33)
esophagitis	3 (10.34)	1 (2.33)	1 (3.45)	0
Abnormal liver function	7 (24.14)	9 (20.93)	2 (6.90)	3 (6.98)
Decreased white cell count	21 (72.41)	30 (69.77)	3 (10.34)	6 (13.95)
Decreased neutrophil count	15 (51.72)	15 (34.88)	7 (24.14)	9 (20.93)
Anaemia	8 (27.59)	11 (25.58)	(3.45)	2 (4.65)
Decreased platelet count	6 (20.69)	9 (20.93)	2 (6.90)	4 (9.30)
Immune-related thyroiditis	2 (6.90)	3 (6.98)	0	0

ES-SCLC, extensive-stage small cell lung cancer; cTRT, consolidative thoracic radiotherapy.

## Discussion

Given the lack of available randomized controlled clinical study data pertaining to the efficacy and safety of cTRT in the era of chemoimmunotherapy, the role of cTRT in ES-SCLC remains unclear. Currently, the quality of the evidence in ASTRO, NCCN, ESMO, and other guidelines regarding the application of cTRT in ES-SCLC patients who respond to chemoimmunotherapy is based on expert opinion. This uncertainty behind these recommendations also reflects the fact that TRT’s benefits are not yet known. It has been reported most recently that several retrospective studies were conducted, but with conflicting results [17–21]. As an example, Li et al. found that cTRT did not improve survival for ES-SCLC treated with first-line chemoimmunotherapy [17]. However, Longo et al. provided not only preclinical data supporting the RT-induced anti-tumor immune response hypothesis, but the clinical data also showed that cTRT was associated with significantly prolonged PFS, as well as a trend toward improved OS [21]. We report the real-world outcomes of cTRT for ES-SCLC patients after initial chemoimmunotherapy at four cancer centers.

In our study, cTRT significantly prolonged mPFS (11.50 months versus 8.02 months, *p* = 0.043) and mOS (28.68 months versus 16.30 months, *p* = 0.033). This may provide further clinical evidence of the biological mechanism of radiation as an immunomodulatory agent, relying on the activation of adaptive and innate immunity to reverse tumor immune desertification [22]. The mPFS and mOS were both longer in the non-CTRT group than in several prospective ES-SCLC trials, such as the IMpower133 trial (5.3 and 13.9 months, respectively) [6]. The reason for this may be that the patients enrolled in this study responded to initial chemoimmunotherapy, rather than the patients in prospective ES-SCLC trials who were newly diagnosed and not receiving treatment. In the subgroup analysis of our study, patients without extrathoracic metastasis or number of initial distant metastases <3 could benefit more from cTRT, which was similar to the further analysis of the CREST trial, where patients with non-liver metastasis or less distant metastases could benefit more from cTRT [23].

Previous studies indicated that immunotherapy combined with TRT might make patients more susceptible to pneumonia [24]. Therefore, the safety of cTRT following chemoimmunotherapy remains a crucial and urgent issue that needs further investigation. Our study had an acceptable and manageable safety profile. The incidence of treatment-related AEs did not increase significantly with the addition of cTRT, and the incidence of pneumonia and grade 3 or higher AEs was low. All patients improved after symptomatic treatment, indicating that toxicity in this study was manageable. A Phase II trial also demonstrated an acceptable safety profile of SHR-1316 (programmed cell death-ligand 1 antibody) in combination with chemotherapy and sequential TRT as first-line treatment for ES-SCLC [25].

A future strategy with enormous potential is the combination of immunotherapy and radiotherapy. However, there are still many challenges that need to be addressed in both the fundamental and clinical fields. Such as the timing of radiotherapy (concurrent or sequential treatment; induction or consolidation), optimal dose and fractionations (standard fractionation, hypofraction, stereotactic body radiotherapy), target volume (including or excluding elective nodes or tumor microenvironment), and potential biomarkers to predict efficacy and toxicity. The ongoing prospective randomized NRG LU007 trial (RAPTOR Trial, NCT04402788) is exploring the combination of consolidative low-dose radiotherapy and atezolizumab in patients with ES-SCLC. The results of our study provide a supportive clinical signal for the conduct of prospective trials, such as the RAPTOR (NCT04402788) trial. Results of this trial are eagerly anticipated.

There are merits to this study. To begin with, this study thoroughly compared cTRT with non-cTRT on survival, toxicity, and confirmed the feasibility of combining cTRT with immunotherapy for ES-SCLC. Secondly, PCI for ES-SCLC after immunotherapy has not been proven to be safe and effective and further research is needed. In this study, PCI was not associated with a significant improvement in OS for patients with ES-SCLC based on univariate analysis. The rate of brain metastasis and neurocognitive toxicity need to be further analyzed in prospective randomized trials. Moreover, we analyzed the failure patterns of the two groups in our study.

Despite the study’s strengths, there are a few limitations. Firstly, this was a retrospective study with a relatively small sample size. Although the median number of immunotherapy cycles received by the two groups was similar, the study includes patients who received different immunotherapy agents (anti-PD-L1 and anti-PD-1). Patients enrolled in the study were inevitably subject to selection bias and heterogeneity. Secondly, as the baseline characteristics were balanced and there was low statistical power from propensity score matching due to the small sample size, propensity score matching was not used to minimize selection bias. Thirdly, the results of subgroup analyses should be interpreted with caution, due to their insufficient statistical power. Finally, subgroups of specific organ metastases are lacking.

## Conclusion

First-line chemoimmunotherapy combined with cTRT is safe and significantly improves survival in ES-SCLC patients. Further studies of this regimen are warranted.

## Supplementary Material

Supplementary table S2.docx

Supplementary table S1.docx

## Data Availability

The data that support the findings of this study are available from the corresponding author upon reasonable request.
