# Supplementary material for: Consolidative thoracic radiotherapy improves the prognosis of extensive stage small-cell lung cancer patients in the chemoimmunotherapy era: a multicenter retrospective analysis
Source: Ann Med. 2025 Aug 4;57(1):2542434. doi: 10.1080/07853890.2025.2542434 (PMC12322984; doi:10.1080/07853890.2025.2542434)
Supplement: Supplementary table S2.docx [file IANN_A_2542434_SM7857.docx]

**Supplementary Table S2** Incidence of other low-grade treatment-related adverse events in ES-SCLC patients.

| Event, *n* (%) | All grades | | Grade 3 or higher | |
| --- | --- | --- | --- | --- |
|  | cTRT  (*n* = 29) | Non-cTRT  (*n* = 43) | cTRT  (*n* = 29) | Non-cTRT  (*n* = 43) |
| Fatigue | 9 (31.03) | 11 (25.58) | 0 | 0 |
| Nausea or vomiting | 5 (17.24) | 8 (18.60) | 0 | 0 |
| Increase of creatinine | 3 (10.34) | 5 (11.63) | 0 | 0 |
| Radiation dermatitis | 1 (3.45) | 0 | 0 | 0 |
| Rash | 2 (6.90) | 3 (6.98) | 0 | 0 |
| Diarrhea | 1 (3.45) | 2 (4.65) | 0 | 0 |

Abbreviations: ES-SCLC, extensive-stage small cell lung cancer; cTRT, consolidative thoracic radiotherapy.
