# Supplementary material for: Consolidative thoracic radiotherapy improves the prognosis of extensive stage small-cell lung cancer patients in the chemoimmunotherapy era: a multicenter retrospective analysis
Source: Ann Med. 2025 Aug 4;57(1):2542434. doi: 10.1080/07853890.2025.2542434 (PMC12322984; doi:10.1080/07853890.2025.2542434)
Supplement: Supplementary table S1.docx [file IANN_A_2542434_SM7856.docx]

**Supplementary table S1** Summary of local response to cTRT.

| Variable | cTRT (*n* = 29) |
| --- | --- |
| Complete response, n (%) | 2 (6.90) |
| Partial response, n (%) | 9 (31.03) |
| Stable disease, n (%) | 13 (44.83) |
| Progressive disease, n (%) | 3 (10.34) |
| Not assessed | 2 (6.90) |

Abbreviations: cTRT, consolidative thoracic radiotherapy.
